# Safety and immunogenicity of boosting with a severe acute respiratory syndrome coronavirus 2 omicron variant mRNA vaccine in healthy adults: An open‐label, and single‐arm Phase 1 study

**DOI:** 10.1002/ctm2.1387

**Published:** 2023-08-23

**Authors:** Suad Hannawi, Rong‐Rong Zhang, Alaa Abuquta, Linda Safeldin, Aala Hassan, Ahmad Alamadi, Tasmiah Hossain, Mohamed Mostafa, Yasser Ghoneim, Jennifer Solo, Hong‐Xia Zheng, Ding‐Feng Wu, Dan‐Dan Yu, Jia‐Cheng Yuan, Di Zhao, Rui Lin, Bo Ying, Cheng‐Feng Qin

**Affiliations:** ^1^ Internal Medicine Department Al Kuwait‐Dubai Hospital Emirates Health Services (EHS) Ministry of Health and Prevention Dubai United Arab Emirates; ^2^ State Key Laboratory of Pathogen and Biosecurity Beijing Institute of Microbiology and Epidemiology Academy of Military Medical Sciences (AMMS) Beijing China; ^3^ Accident and Emergency Department Al Kuwait‐Dubai Hospital EHS Ministry of Health and Prevention Dubai United Arab Emirates; ^4^ General Surgery Department Al Kuwait‐Dubai Hospital EHS Ministry of Health and Prevention Dubai United Arab Emirates; ^5^ PDC FZ‐LLC Contract Research Organization Dubai United Arab Emirates; ^6^ Abogen Biosciences Suzhou Abogen Biosciences Co., Ltd. Suzhou China

Dear Editor,

The coronavirus disease 2019 (COVID‐19) caused by severe acute respiratory syndrome coronavirus 2 (SARS‐CoV‐2) continues to be prevalent worldwide. The development and global deployment of SARS‐CoV‐2 vaccines have reduced the burden of COVID‐19.^1^ However, the continuous mutation of SARS‐CoV‐2 and waning immunity led to decreased effectiveness of COVID‐19 vaccines in a real‐world setting. On November 26, 2021, the World Health Organization (WHO) announced the Omicron variant to be the fifth Variant of Concern,^2^ and the development of Omicron BA.1 specific vaccine represents high priority. Thus, boosting with a variant‐targeting vaccine represents a more promising strategy. Moderna and Pfizer/BioNTech have updated their bivalent mRNA vaccine to contain mRNAs encoding the full S protein of Omicron variants.^3^, ^4^

Previously, we have developed a potent COVID‐19 mRNA vaccine (termed ARCoV) targeting the receptor‐binding domain (RBD) of the prototype strain of SARS‐CoV‐2, that has been licensed for emergency use in Indonesia.^5^, ^6^ In response to the emergence of the Omicron variant, we have also developed an updated version of ARCoV, which encodes the RBD of the S protein of Omicron BA.1 sub‐lineage (B.1.1.529) (Figure S1).^7^ Preclinical studies showed that the updated Omicron BA.1 targeting mRNA vaccine (named ABO1009‐DP) exhibited potent immunogenicity in mice.^7^ Herein, we report the results from an open‐label and single‐arm Phase 1 study (NCT05433194) to evaluate the safety and immunogenicity of 15 μg of ABO1009‐DP as the booster dose in adults.

A total of 48 participants who had received two or three doses of inactivated COVID‐19 vaccines were enrolled from 277 volunteers (Figure S2). All these participants were between 18 and 59 years old with a mean age of 26.1 years. Forty‐seven participants were male. Forty‐six participants were Asian, and two participants were African American. Safety data were observed 28 days after the boost in all participants (Table S1). In total, 23 (47.9%) participants reported solicited adverse reactions (ARs) within 7 days after the booster vaccination. According to the China National Medical Products Administration (NMPA) criteria, all ARs were mild, and no Grade 3 or Grade 4 solicited adverse events (AEs) were reported (Table 1 and Table S2). The pain was the only locally solicited AR reported by 11 (22.9%) participants. The most common systemic solicited ARs were headache (20.8%) and fever (18.8%) (Figure S3). Only one (2.1%) participant reported a Grade 2 fever (38.0–38.5°C, axillary), all other fever events were reported as Grade 1 (37.3–38.0°C, axillary) (eight participants, 16.7%). The median duration of solicited ARs was 2.0 days. All solicited ARs were resolved within 7 days after a boost, except that one participant had a cough that lasted for 8 days.

**TABLE 1:** Solicited adverse reactions within 7 days, unsolicited adverse events until Day 28, graded by National Medical Products Administration (NMPA) criteria.

Adverse reactions/events, *n* (%)	ABO1009‐DP, *N* = 48
**Any Adverse Events, *n* (%)**	
Any	35 (72.9)
Related to vaccination	35 (72.9)
**Solicited adverse reactions within 7 days**	
Any	23 (47.9)
**Local adverse reactions**	
Any	11 (22.9)
Injection site pain	11 (22.9)
Induration	0
Swelling	0
Redness	0
Pruritus	0
Rash	0
Cellulitis	0
**Systemic adverse reactions**	
Any	19 (39.6)
Headache	10 (20.8)
Fever	9 (18.8)
Fatigue	2 (4.2)
Cough	1 (2.1)
Nausea	0
Vomiting	0
Muscle pain	0
Anorexia	0
Diarrhea	0
Acute allergic reactions	0
Dyspnea	0
Abnormal cutaneous mucosa	0
**Unsolicited adverse events until Day 28** ^a^	
Any	26 (54.2)
Feeling hot^b^	16 (33.3)
Pain	5 (10.4)
Blood triglycerides increased	5 (10.4)
Pruritus	3 (6.3)
Back pain	2 (4.2)
Headache	2 (4.2)
Medically attended adverse events (MAAEs)	4 (8.3)
Chest pain	2 (4.2)
Cough	2 (4.2)
MAAE related to vaccination	3 (6.3)
**Serious Adverse Events**	
Any	0
**Adverse Events of Special Interest**	
Any	0

*N* indicates the number of participants in the specified group, and *n* indicates the number of participants with the occurrence of the specified adverse event category. The rows of 'any’ events show the number and percentage of participants who reported at least one occurrence of corresponding events.

^a^ The unsolicited adverse events reported by more than one participant were included.

^b^ Feeling hot described the participants who felt the body temperature increase, but the measured temperature did not reach 37.3°C which was defined as Grade 1 fever by NMPA grading criteria.

Unsolicited AEs within 28 days after the booster dose was reported by 26 (54.2%) participants. The most frequently unsolicited AEs were feeling hot, pain and blood triglycerides increased. No serious adverse event (SAE), AEs of special interest (AESI), or AE leading to study discontinuation was reported up to 28 days after the booster vaccination (Table 1). In general, the ABO1009‐DP booster for up to 28 days was safe and well‐tolerated. The majority of the reported events were mild and local and systemic solicited ARs were transient. In general, our safety findings about ABO1009‐DP supported that this vaccine's safety profile was similar to the results of other mRNA vaccines in terms of the reported category of adverse events and the severity.^8^

Live virus nAb titers have been well deemed as the surrogate of protection.^5^, ^9^ Herein, the plaque‐reduction neutralization testing (PRNT) assay was performed using an Omicron variant strain G42‐21‐NRHMB0032 according to guidelines from WHO.^10^ Sera from all participants (*n* = 48) were collected at days 0 and 14 post booster. All participants developed nAbs against Omicron BA.1 on Day 14, and the geometric mean titer (GMT; 95% CI) was calculated to 1580.0 (95% confidence interval [CI]: 1104.0–2261.0) (Figure 1A). Compared with the GMT at Day 0, the booster yielded a geometric mean fold rise (GMFR) of 11.5 (95% CI: 8.31–16.02), which is comparable with other Omicron component mRNA vaccines.^3^ The GMFR of nAbs induced by mRNA‐1273.214 (Moderna) and BA.1‐adapted BNT162b2 (Pfizer/BioNTech) was 8.0‐ and 13.5‐fold, respectively.^3^, ^8^ The seroconversion rate of the live virus nAb on Day 14 was 93.8% (95% CI: 82.8%−98.7%). Also, sub‐group analysis showed that GMTs of nAbs of participants who had completed a three‐dose were higher than that who received two‐dose primary vaccination (Figure 1B). Additionally, ELISpot assays were performed using peripheral blood mononuclear cells (PBMCs) from 30 participants at days 0 and 14 post‐booster. ABO1009‐DP induced a 5.2‐ and 3.0‐fold rise in the geometric mean of interferon‐γ^+^ (IFN‐γ^+^) against the wild‐type variant and Omicron BA.1 variant, respectively (Figure 1C).

**FIGURE 1:**
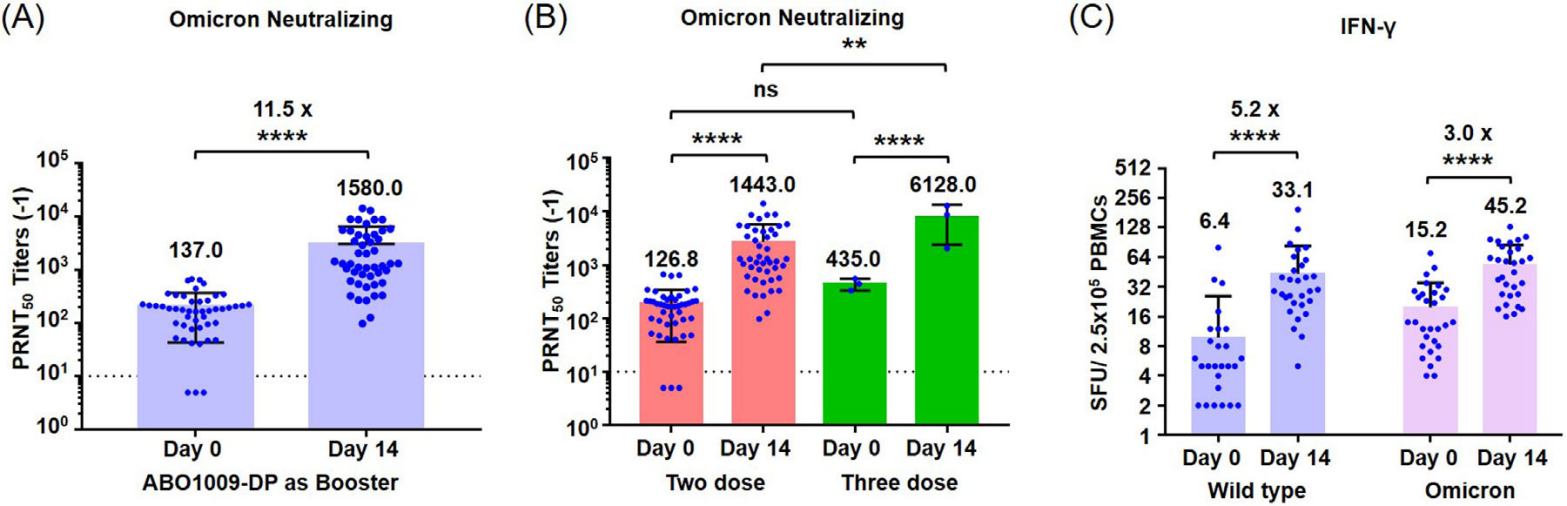
Immunogenicity of the booster with ABO1009‐DP in healthy adults. (A) The dots show the 50% Plaque reduction neutralization test (PRNT_50_) results against the Omicron BA.1 variant from individual serum samples at baseline and Day 14 after a booster dose of ABO1009‐DP (n = 48). The bars show geometric mean titer (GMT) with 95% CI. For values below the lower limit of quantification (LLOQ) = 10, LLOQ/2 values were plotted. Seroconversion rates are shown at the top of the figure. (B) The nAb titers of the booster with ABO1009‐DP in healthy adults who had completed a two‐dose (*n* = 45) or three‐dose (*n* = 3) primary vaccination. (C) The numbers of IFN‐γ secreting T cell responses were assessed with spots in direct ex vivo IFN‐γ ELISpot with Spike Protein Pool of Omicron BA.1 variant and wild‐type variant stimulus at baseline (*n* = 30) and Day 14 (*n* = 30) after a booster dose of ABO1009‐DP. The bars show GMT with 95% CI. Ns, not significant, **p < 0.01, ****p < 0.0001.

This study has several limitations. First, data interpretation is based on a small sample size with an unbalanced ratio between race and sex. Secondly, Omicron variant BA.1 has continued to evolve into new sub‐lineages, including BA.2, BA.4‐BA.5, BA.4.6, BA.2.75.2, BQ.1.1 and XBB.1, most of which showed significant immune escape capability from previous variants. The neutralization activity against these omicron sub‐lineages is yet to be carried out.

In summary, ABO1009‐DP demonstrated a good safety profile and well tolerability administered as a booster vaccination. As an Omicron‐specific mRNA vaccine, a single dose of ABO1009‐DP booster induced excepted humoral and cellular immunity against Omicron variant BA.1. The findings of this study support further clinical trials to assay the efficacy of ABO1009‐DP.

## CONFLICT OF INTEREST STATEMENT

B.Y. is the founder of Abogen Bioscience. H.‐X.Z., D.‐F.W., D.‐D.Y., J.‐C.Y., D.Z., and R.L., are employees of Abogen Bioscience. The other authors declare no conflict of interest.

## FUNDING INFORMATION

This work was supported in part by the National Key Research and Development Project of China (2021YFC2302400 and 2021YFC0865800). This study was funded by Abogen Biosciences.

## Supporting information

Supporting Information

## Data Availability

On account of this clinical trial is ongoing, participant‐level data and supporting clinical documents may be available upon request after the trial is complete. Such requests can be made to the corresponding authors upon request. To gain access, data requesters will need to sign a data access agreement.
