# Supplementary material for: Safety and immunogenicity of boosting with a severe acute respiratory syndrome coronavirus 2 omicron variant mRNA vaccine in healthy adults: An open‐label, and single‐arm Phase 1 study
Source: Clin Transl Med. 2023 Aug 23;13(8):e1387. doi: 10.1002/ctm2.1387 (PMC10447877; doi:10.1002/ctm2.1387)
Supplement: Supplementary file 1 — Supporting Information [file CTM2-13-e1387-s001.docx]

**Supplementary Information**

**Materials and Methods**

**Study design and participants**

This Study (registered with ClinicalTrials.gov, NCT05433194) was an open-label and single-arm study for sequential immunization in vaccinated participants aged 18 years and older to evaluate the safety, tolerability, and immunogenicity of ABO1009-DP administered as a booster dose. The sample size of 48 participants was determined based on the clinical and practical considerations to meet the study objective.

All enrolled participants had completed a two-dose or three-dose primary vaccination 3-12 months before the screening. All these participants were between 18 and 59 years old with a mean age of 26.1 years. Forty-seven participants were male. Forty-six participants were Asian, and two participants were African American. At the screening visit, all participants were tested for the SARS-CoV-2 reverse transcription-polymerase chain reaction (RT-PCR) via a nasopharyngeal nucleic acid diagnostic test (Biogenix lab), and participants with positive results were excluded. Based on medical history, medication history, physical examination, vital signs, and laboratory tests, all enrolled participants were in good health. All participants with fever (Participants with axillary temperature or equivalent ≥ 37.3℃ at screening or within 72 hours) were excluded. Participants with serious or uncontrolled diseases, such as nervous system disorders, cardiovascular disorders, liver and kidney disorders, respiratory system disorders, blood and lymphatic system disorders, metabolism and skeletal disorders, etc. were excluded. Pregnant or breastfeeding female participants were excluded.

**Vaccine and vaccination**

ABO1009-DP is a modified SARS-CoV-2 variant (Omicron) messenger ribonucleic acid (mRNA) vaccine based on the first-generation vaccine of AWcorna, which has been granted the EUA in Indonesia on September 30, 2022. The active ingredient of ABO1009-DP is mRNA encoding SARS-CoV-2 Omicron variant B.1.1.529 spike protein (S protein) receptor-binding domain (RBD) antigen. Compared with the original vaccine AWcorna, the modified vaccine ABO1009-DP encodes the S-RBD sequence closely resembles that of the prototype antigen, with only 15 amino acids mutations. The antigen sequence alignment of AWcorna and ABO1009-DP is displayed in Figure S1. This vaccine is manufactured by encapsulating the respective variant S-RBD antigen mRNA with lipid nanoparticles (LNP) vector delivery system as previously described^1^. A single dose of ABO1009-DP contained 15 ug of mRNA. All enrolled participants were injected with one dose of ABO1009-DP intramuscularly in the lateral deltoid region of the upper arm on Day 0.

**Outcomes**

The safety of ABO1009-DP in vaccinated healthy adults was the primary objective in this trial. Incidence of solicited adverse events (AEs) from Day 0 to Day 7, unsolicited AEs from Day 0 to Day 28, AEs from abnormal laboratory values in blood routine, blood coagulation function, blood biochemistry, and urinalysis indicators from Day 0 to Day 4 were the primary endpoints for safety evaluation. All abnormal lab values will be reported as AEs.

The secondary objectives were to evaluate the humoral and cellular immunity and safety of ABO1009-DP in vaccinated healthy adult participants. The secondary immunogenicity endpoints included live-virus nAbs at Day 14 after vaccination, seroconversion rate of the live-virus nAb at Day 14 after vaccination, and the number of T cells secreting cytokines interferon-γ (IFN-γ) at Day 14 after vaccination. Seroconversion was defined as a fourfold or great increase if the nAb titers were above the lower limit of quantification (LLOQ) before the booster, or a variation from below the LLOQ to above the LLOQ. The secondary safety endpoints were serious adverse events (SAEs), adverse events of special interests (AESIs), and medically attended AEs (MAAEs) through 12 months after the booster vaccination. In this manuscript, the primary safety results (28-day observation) and preliminary immunogenicity results (14-day observation) were included. Data and Safety Monitoring Board (DSMB) reviewed safety and immunogenicity data to ensure the participant’s safety and provided suggestions to the sponsor on whether to continue, change, or terminate the clinical trial.

**Safety assessment**

Solicited local AEs (injection site pain, induration, swelling, redness, pruritus, rash, and cellulitis), solicited systemic AEs (fever, headache, cough, fatigue, nausea, vomiting, muscle pain, anorexia, diarrhea, acute allergic reactions, dyspnea, and abnormal cutaneous mucosa), and unsolicited AEs were collected 7 and 28 days after the vaccination, respectively. All these AEs were self-reported by the participants and were verified by investigators via a diary card issued on Days 0, 7, 14, and 28 after the vaccination. AEs related to abnormal laboratory values of blood routine, blood coagulation function, blood biochemistry, and urinalysis indicators were collected 4 days after the vaccination.

The SAEs, MAAEs, and AESIs will be monitored for 12 months after one dose of vaccination. All participants will receive safety follow-up via telephone/e-mail 28 days after finishing the vaccination, once every 4 weeks in the first 3 months, and every 8 weeks (± 3 days) afterward until 12 months after vaccination. The long-term safety data will be reported in the future.

The adverse events were graded according to the Guidelines for Grading Standard of Adverse Events in Clinical Trials of Preventive Vaccines by the China National Medical Products Administration (NMPA) (Version 2019).

**Laboratory assays**

Biogenix Lab was one of the first laboratories with a massive throughput testing service to address the need for population scale detection and diagnosis of COVID-19 in UAE (United Arab Emirates). It was CAP and ISO15189 accredited, located in Abu Dhabi, UAE.

The nAbs in sera against the Omicron variant strain G42-21-NRHMB0032 were determined by 50% plaque reduction neutralization test (PRNT_50_) assay. Serum samples were two-fold serially diluted with an initial serum dilution of 1:10 and were incubated at 37°C for 1 hour with an equal volume of the virus stock contain approximately 100 PFU of virus per well. Each diluted sample/viral mixture were then applied to Vero E6 cells culture monolayers in 6 well plates and incubated at 37°C for 2 hours. The media were gently aspirated out of the infected monolayer well and discarded. Cells were overlaid with 2 mL of the 2.5% low melting point agarose/media mixture and then incubated at 37°C, 5% CO_2_ for 3-5 days. The infected cells were fixed with 4% formaldehyde, followed by staining with 0.5% crystal violet solution. The plate was counted using the colony counter for the number of plaques. The PRNT_50_ titers were calculated by the method of Spearman–Karber.

Cellular immune responses in the 30 vaccinated participants were evaluated by using Enzyme-linked immunospot (ELISpot) assay. Peripheral blood mononuclear cells (PBMCs) were collected and the IFN-γ secreting T-cell responses were performed in vitro with the T-SPOT®. COVID test kit (Oxford Immunotec, COV.435/200) with Omicron BA.1 spike protein (Genscript, RP30219) and T SPOT spike Ag from wild-type strain included in the kit. Briefly, the PBMCs of the participants were plated at 250,000 cells per well and stimulated using the Omicron BA.1 spike protein (Genscript, RP30219), T SPOT spike Ag from wild-type strain, positive control solution or AIM-V cell culture medium as negative control, respectively. After incubation at 37 °C for 16-20 hours, the plate was washed with 1x PBS solution and working strength Conjugate Reagent solution was added to each well followed by incubation at 2-8°C for one hour. The conjugate was discarded and the plate was washed with 1x PBS solution. Then the plate incubated at room temperature for 7 min after the Substrate solution added to each well. Finally, Reaction terminated with distilled water. After the plate dries, the number of dark blue spots were counted and recorded.

**Statistical analysis**

This study was an open-label and single-arm study, and no randomization and blinding were applied. No hypothesis testing was performed.

The safety and immunogenicity of the one-dose booster were descriptively summarized. The sample size was based on clinical and practical considerations and considered sufficient to support the descriptive summary.

Safety endpoints are described as frequency (%) of the adverse reactions (ARs) or adverse events (AEs) during the observation period. Immunogenicity data was analyzed in the modified Intention-to-Treat set, including all participants who received one-dose booster and had the results of immunogenicity at baseline and after vaccination. The seroconversion rate of anti-SARS-CoV-2 Omicron variant live virus nAbs, and its 95% confidence interval on Day 14 following the vaccination were calculated. The live-virus nAb titers against Omicron variant was calculated using geometric mean titers (GMTs) and its 95% confidence interval. GMT, Geometric mean fold rise (GMFR) and 95% confidence interval were calculated by a naïve approach that the limits of the confidence interval are back-transformed.

SAS v9.4 or higher version was used to perform the statistical analyses.


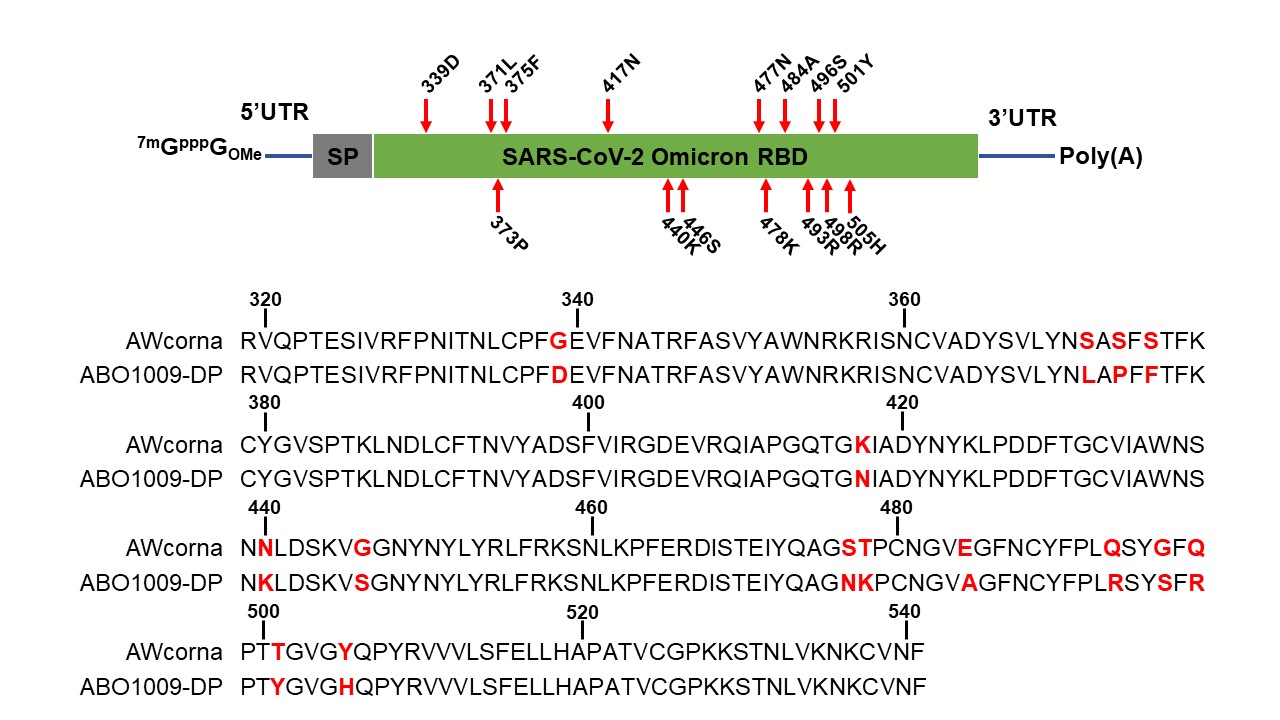


**Supplementary Figure 1** Schematic representation of the mRNA construct encoding Omicron RBD and amino acid sequence alignment of the full RBD of AWcorna and ABO1009-DP. The mutation sites were indicated with the red arrows and amino acids of variant mutations are marked in bold red font.


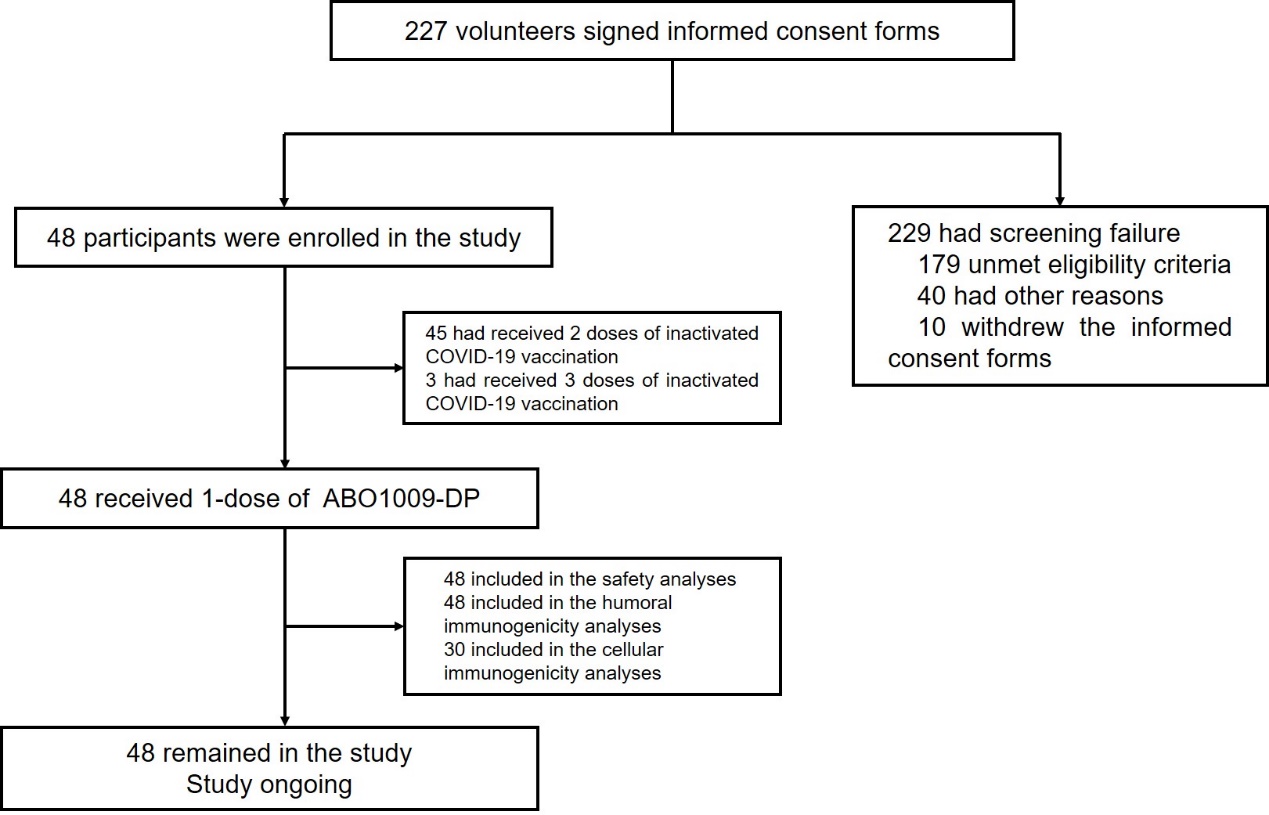


**Supplementary FIGURE 2** Trial flow diagram. The study is ongoing when writing this manuscript.

**
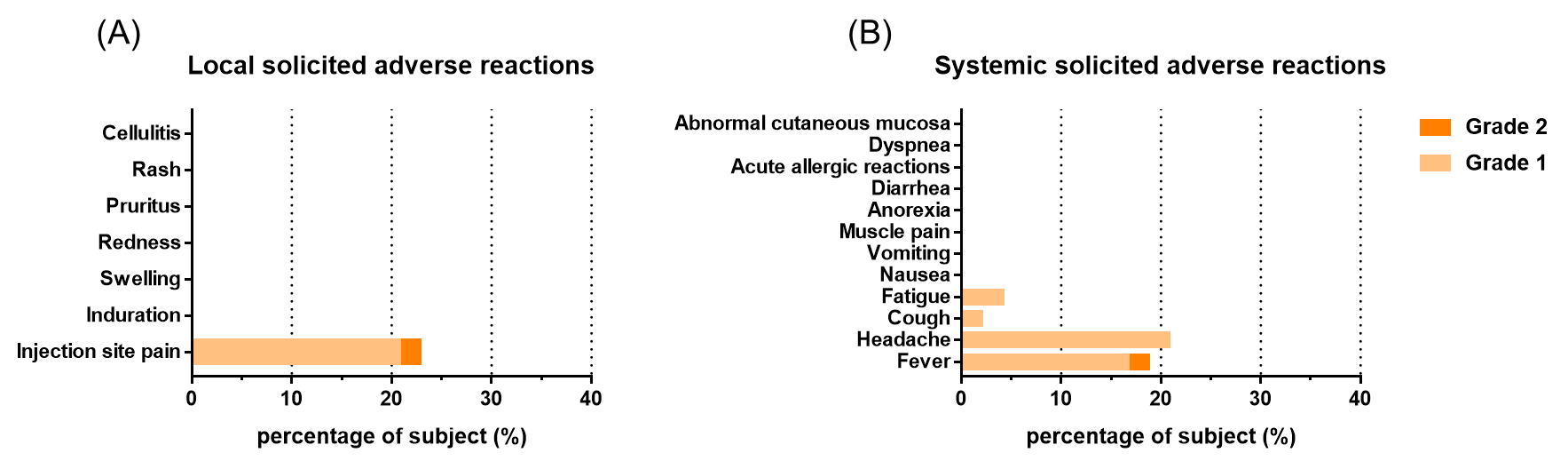
**

**Supplementary FIGURE 3** Safety of the booster with ABO1009-DP in healthy adults. (A) Solicited local adverse reactions in 7 days after the booster vaccination, graded by NMPA criteria. (B) Solicited systemic adverse reactions in 7 days after the booster vaccination, graded by NMPA criteria.**Supplementary Table 1** Demographics and characteristics of all enrolled participants.

| **Characteristic (%)** | **ABO1009-DP**  **1-dose booster**  **n=48** |
| --- | --- |
| **Age (year), mean (range)** | 26.1 (18-42) |
| 18~59 years, n (%) | 48 (100) |
| **Sex** |  |
| Male | 47 (97.9) |
| Female | 1 (2.1) |
| **Race, n (%)^a^** |  |
| Asian | 46 (95.8) |
| African American | 2 (4.2) |
| **Body-mass index (kg/m^2^)** |  |
| Mean (SD) | 22.5 (3.32) |
| **SARS-CoV-2 RT-PCR Test Result at baseline, n (%) ^b^** |  |
| Negative | 48 (100) |

^a^ Race was reported by the participants.

^b^ Baseline SARS-CoV-2 status was negative if there were a negative RT-PCR test (Biogenix).

**Supplementary Table 2** Toxicity grading scales for solicited local and systemic adverse events by National Medical Products Administration (NMPA).

|  | **Grade 1** | **Grade 2** | **Grade 3** | **Grade 4** |
| --- | --- | --- | --- | --- |
| **Local adverse events** | | | | |
| Injection site pain | No influence or mild influence on physical activity | Influence on physical activity | Influence on daily activity | Loss of basic self-care ability, or hospitalization |
| Induration*, swelling (alternative)** # | Diameter of 2.5 - < 5 cm or area of 6.25 - < 25 cm^2^, and no influence or slight influence on daily activity | Diameter of 5 - < 10 cm or area of 25 - < 100 cm^2^, or influence on daily activity | Diameter of ≥ 10 cm or area of ≥ 100 cm^2^ or ulceration or secondary infection or phlebitis or aseptic abscess or wound drainage or severely influence on daily activity | Abscess, exfoliative dermatitis, dermis or deep tissue necrosis |
| Skin rash*, redness (alternative)** # | Diameter of 2.5 - < 5 cm or area of 6.25 - < 25 cm^2^, and no influence or slight influence on daily activity | Diameter of 5 - < 10 cm or area of 25 - < 100 cm^2^, or influence on daily activity | Diameter of ≥ 10 cm or area of ≥ 100 cm^2^ or ulceration or secondary infection or phlebitis or aseptic abscess or wound drainage or severely influence on daily activity | Abscess, exfoliative dermatitis, dermis or deep tissue necrosis |
| Pruritus | Vaccination site pruritis, relieved spontaneously or within 48 h after treatment | Vaccination site pruritis, not relieved within 48 h after treatment | Influence on daily activity | NA |
| Cellulitis | NA | Non-injection therapy is required (e.g., oral anti-bacterial, anti-fungal, anti-viral drug therapy) | Intravenous therapy is required (e.g., intravenous anti-bacterial, anti-fungal, antiviral drug therapy) | Sepsis, or tissue necrosis, etc. |
| **Systemic adverse events** | | | | |
| Headache | No influence on daily activity, treatment not indicated | Transient, slight influence on daily activity, treatment or intervention indicated possibly | Severely influence on daily activity, treatment or intervention indicated | refractory, emergency treatment or hospitalization indicated |
| Fever*** (axillary, °C) | 37.3-38.0 | 38.0-38.5 | 38.5-39.5 | ≥39.5 for more than 3 days |
| Fatigue | Affect no daily activity | Affect normal daily activity | Severely affect daily activity and cannot work | Emergency treatment or hospitalization indicated |
| Cough | Transient, treatment not indicated | Persistent cough, treatment is effective | Paroxysmal cough, which cannot be controlled through treatment | Emergency treatment or hospitalization indicated |
| Nausea | Transient (< 24 hours) or intermittent and basically normal food intake | Persistent nausea resulting in food intake decreased (24 - 48 hours) | Persistent nausea resulting in almost no food intake (> 48 hours) or intravenous fluids replacement required | Life-threatening (such as hypotensive shock) |
| Vomiting | 1~2 times/24 h and not limiting activity | 3~5 times/24 h or limiting activity | > 6 times / 24 hours or intravenous fluids replacement required | Hypotensive shock, with hospitalization or nutrition supply via other routes required |
| Muscle pain  (non-vaccination site) | Affect no daily activity | Slightly influence on daily activity | Severe muscle pains, severely affect daily activity | Emergency treatment or hospitalization indicated |
| Anorexia | Loss of appetite, but no reduction in food intake | Loss of appetite, decreased food intake, but no significant weight loss | Loss of appetite, with significant weight loss | Requiring intervention measures (such as intragastric tube feeding and parenteral nutrition) |
| Diarrhea | Mild or transient, 3~4 stools/day, abnormal stool appearance, or mild diarrhea persisting for less than 1 week | Moderate or persistent, 5~7 stools/day, abnormal stool appearance, or diarrhea >1 week | > 7 times/day, abnormal feces, or bloody diarrhea, orthostatic hypotension, electrolyte imbalance, with > 2 L intravenous infusion required | Hypotensive shock, hospitalization required |
| Acute allergic reactions^****^ | Urticaria localized (blister), treatment not indicated | Local urticaria requiring treatment or mild angioedema requiring no treatment | Extensive urticaria or angioedema, treatment indicated or mild bronchospasm | Allergic shock or life-threatening bronchospasm or laryngeal edema |
| Dyspnea | Exercise‐induced dyspnea | Dyspnea during normal activity | Dyspnea at rest | Dyspnea, requiring oxygen therapy, hospitalization or assistant breathing |
| Abnormal cutaneous mucosa | Erythema/pruritus/color  changed | Diffuse rash/maculopapular rash/drying/desquamation | Blister/effusion/desquamation/ulcer | Exfoliative dermatitis involving mucous membrane, erythema multiforme or suspected Stevens-Johnsons syndrome |

Note: *In addition to assessing directly by measuring diameters, the changes in measurement results should also be recorded. ** Use the maximum measured diameter or area.

# Evaluation and grading of induration and swelling, rash and redness were based on functional level and actual measurement results, the indicators with higher grades are selected.

*** Axillary temperature is usually used in China, which is converted into oral temperature and anal temperature when necessary. Generally, oral temperature = axillary temperature +0.2℃; rectal temperature=axillary temperature + (0.3~0.5℃). When persistent hyperpyrexia occurs, the cause of hyperpyrexia should be identified as soon as possible.

**** Type I hypersensitivity.

**References**

**1.** Zhang NN, Li XF, Deng YQ, et al. A Thermostable mRNA Vaccine against COVID-19. *Cell.* Sep 3 2020;182(5):1271-1283 e1216.
